# High-Density Lipoprotein (HDL) Particle Subpopulations in Heterozygous Cholesteryl Ester Transfer Protein (CETP) Deficiency: Maintenance of Antioxidative Activity

**DOI:** 10.1371/journal.pone.0049336

**Published:** 2012-11-26

**Authors:** Sandrine Chantepie, Andrea E. Bochem, M. John Chapman, G. Kees Hovingh, Anatol Kontush

**Affiliations:** 1 INSERM UMRS 939, Pitie-Salpetriere University Hospital, Paris, France; 2 Department of Vascular Medicine, Academic Medical Center, Amsterdam, The Netherlands; 3 Department of Scientific Research, University Pierre and Marie Curie - Paris 6, Paris, France; 4 AP-HP, Groupe hospitalier Pitié-Salpétrière, Paris, France; Harvard Medical School, United States of America

## Abstract

Cholesteryl ester transfer protein (CETP) deficiency causes elevated high-density lipoprotein-cholesterol (HDL-C) levels; its impact on HDL functionality however remains elusive. We compared functional and compositional properties of HDL derived from 9 Caucasian heterozygous CETP mutation carriers (splice-site mutation in intron 7 resulting in premature truncation) with those of 9 age- and sex-matched normolipidemic family controls. As expected, HDL-C levels were increased 1.5-fold, and CETP mass and activity were decreased by −31% and −38% respectively, in carriers versus non-carriers. HDL particles from carriers were enriched in CE (up to +19%, p<0.05) and depleted of triglycerides (TG; up to −54%, p<0.01), resulting in a reduced TG/CE ratio (up to 2.5-fold, p<0.01). In parallel, the apoA-I content was increased in HDL from carriers (up to +22%, p<0.05). Both the total HDL fraction and small, dense HDL3 particles from CETP-deficient subjects displayed normal antioxidative activity by attenuating low-density lipoprotein oxidation with similar efficacy on a particle mass basis as compared to control HDL3. Consistent with these data, circulating levels of systemic biomarkers of oxidative stress (8-isoprostanes) were similar between the two groups. These findings support the contention that HDL functionality is maintained in heterozygous CETP deficiency despite modifications in lipid and protein composition.

## Introduction

Low circulating levels of high-density lipoprotein-cholesterol (HDL-C) constitute a significant and independent predictor of cardiovascular disease (CVD). This association may reflect deficiency of multiple antiatherogenic activities of HDL particles, including the capacity to act as an acceptor for cellular cholesterol together with antioxidative and anti-inflammatory actions [1].

HDL-C levels are subject to strong genetic control, with heritability ranging between 40% and 60% [2], [3], [4]. Factors determining HDL-C levels can be either of monogenic, polygenic, environmental, or multifactorial nature [5]. The CETP gene is one of the major genes affecting HDL metabolism; the gene codes for cholesteryl ester transfer protein (CETP), a protein responsible for the transfer of cholesteryl ester (CE) from HDL to apolipoprotein (apo) B-containing particles, particularly very low-density lipoprotein (VLDL), in exchange for triglyceride (TG) [6]. As a corollary, CETP deficiency typically results in an antiatherogenic lipid profile characterized by elevated levels of HDL-C and decreased to normal concentrations of low-density lipoprotein-cholesterol (LDL-C) [7], [8].

The CETP gene is highly polymorphic with several common polymorphisms as well as rare mutations [9]. Homozygous CETP deficiency associated with complete loss of CETP activity leads to the accumulation of large, CE-rich HDL and elevation of HDL-C levels up to 5-fold. Such complete CETP deficiency is exceedingly rare in Caucasians, but frequently observed in subjects of Asian origin [10], [11]. Heterozygous deficiency of CETP results in less pronounced increases in HDL-C levels of +10 to +30% [12].

Despite elevated HDL-C levels, data on coronary risk in CETP-deficient subjects are conflicting [13], [14], [15], [16], [17], [18], [19], [20], potentially reflecting defective atheroprotective functionality of HDL in this condition. CETP deficiency does not appear to compromise the capacity of HDL to efflux and transport cholesterol [21], [22], [23]; little is however known of other atheroprotective activities of HDL. To evaluate the effect of CETP deficiency on HDL function, we characterised compositional properties of HDL particles and their antioxidative activity in a hyperalphalipoproteinemic Dutch family with heterozygous CETP deficiency resulting from a splice-site mutation and premature protein truncation. Our findings further support the contention that HDL functionality is conserved in heterozygous CETP deficiency.

## Subjects and Methods

### Subjects

Nine Dutch subjects with heterozygous CETP deficiency due to a splice-site mutation in intron 7 resulting in premature truncation [24] were recruited at the Academic Medical Center, Amsterdam (The Netherlands). Age- and gender-matched family controls with HDL-C levels between the 10th and the 90th percentiles for the general population were recruited at the same center. Subjects displaying severe hypertriglyceridemia (defined as plasma levels of TG >400 mg/dl) were excluded from the study.

### Blood Samples

Venous blood was collected after an overnight fast. Routine biologic analyses were performed within 3 hours of blood sampling following centrifugal isolation of plasma. EDTA plasma and serum were immediately isolated, mixed with sucrose (final concentration 0.6%) as a cryoprotectant for lipoproteins [25], and frozen at −80°C under nitrogen until using for lipoprotein analysis (see below). Total cholesterol (TC) and TG concentrations were determined by automated enzymatic methods (Konelab, Thermoclinical Labsystems, Cergy Pontoise, France, and Biomerieux, Marcy L’Etoile, France, respectively). HDL-C was determined by a direct method using Konelab (Thermo Scientific, Waltham, MA, USA) [26]. LDL-C was calculated using Friedewald’s equation [27]. Exogenous plasma CETP activity was determined using the Roar Biomedical kit (NY, New York) in which intra- and inter-assay coefficients of variation were less than 3% [28]. Plasma CETP concentration was determined by ELISA [29].

### Isolation of Lipoproteins

Lipoproteins were fractionated by single-spin equilibrium density gradient ultracentrifugation [30] for 48 h at 40,000×g in a SW41-Ti rotor (Beckman Coulter, Pasadena, CA, USA) at 4°C. Five HDL subfractions were isolated: large, light HDL2b (density 1.063–1.087 g/ml) and 2a particles (density 1.088–1.110 g/ml), and small dense HDL3a (density 1.110–1.129 g/ml), 3b (density 1.129–1.154 g/ml) and 3c particles (density 1.154–1.170 g/ml) [30]. Reference LDL was isolated using the same procedure from one healthy, normolipidemic donor for all oxidation experiments. After dialysis against Dulbecco’s PBS (pH 7.4) for 24 h at 4°C to remove EDTA and KBr, lipoproteins were maintained at 4°C and used within 1 week.

### Characterisation of Lipoproteins

Total cholesterol (TC), free cholesterol (FC), phospholipid (PL) and TG concentrations were measured using commercially available kits (CHOP-PAP, Biomerieux, France). CE content was calculated by multiplying the difference between TC and FC by 1.67 [30]. Total protein was measured using the BCA assay. Total lipoprotein mass was calculated as the sum of the mass of total protein, CE, FC, PL and TG. Apolipoproteins apoA-I and apoA-II were quantified by immunonephelometry [31].

### Enzymatic Activities of HDL Subpopulations

PON1 activity was determined photometrically at 270 nm as arylesterase activity using phenyl acetate as a substrate [31]. Inter- and intra-assay coefficients of variation were 2.2 and 9.8%, respectively.

### Antioxidative Activity of HDL3b and HDL3c

Reference LDL (TC, 0.26 mmol/l, equivalent to 10 mg/dl) was incubated at 37°C in PBS in the presence of a water-soluble azo-initiator of oxidation, 2,2′-azo-bis-(2-amidinopropane) hydrochloride (AAPH; 1 mmol/l). Individual HDL subpopulations (10 mg/dl) or total HDL (40 mg total mass/dl) were added to LDL directly before oxidation on a total mass basis as previously described [31]. Total HDL from each donor was prepared by mixing all five HDL subfractions at their equivalent serum concentrations. The PBS was treated with Chelex 100 ion exchange resin (BioRad, Marnes-la-Coquette, France) for 1 h to remove contaminating transition metal ions. Accumulation of conjugated dienes was continuously measured as the increment in absorbance at 234 nm [31]; two characteristic phases were identified, the lag phase and the propagation phase. To characterize the oxidation kinetics, average oxidation rate and the duration of propagation phase were calculated for each absorbance curve as described elsewhere [31].

### Plasma Markers of Oxidative Stress

Systemic levels of oxidative stress were assessed as plasma levels of oxidised LDL (Mercodia, Uppsala, Sweden) and of 8-isoprostanes, products of non-enzymatic oxidation of arachidonic acid in vivo that represent a robust marker of systemic oxidative stress [32], by a commercial ELISA assay after purification (Cayman Chemical, Ann Arbor, MI, USA; inter- and intra-assay coefficients of variation 8.0 and 11.1%, respectively).

### Statistical Analysis

Between-group differences were analysed by Student’s two-tailed t-test for independent groups. All results are expressed as means ± SDs. A p value of less than 0.05 was considered statistically significant.

## Results

### Characteristics of Subjects

Biological and clinical characteristics of subjects are reported in Table 1. The CETP-deficient group did not differ from the non-carrier family control group in terms of age, BMI and plasma levels of total cholesterol, TG, non-HDL-C and LDL-C. As expected, HDL-C levels were increased 1.5 -fold while CETP mass and activity were decreased by −31% and −38% respectively in the CETP-deficient group as compared to controls.

**Table 1:** Biological and clinical characteristics of subjects.

	CETP-deficient subjects (n = 9)	Family control subjects (n = 9)
Sex (m/f)	5/4	5/4
Age (years)	55.0±16.3	54.3±14.0
Body mass index (kg/m^2^)	26.1±3.3	26.1±5.6
Total cholesterol (mg/dl)	240±33	220±47
TG (mg/dl)	137±80	158±82
Non-HDL-C (mg/dl)	166±49	170±40
LDL-C (mg/dl)	140±33	141±33
HDL-C (mg/dl)	**75**±26* (+50%)	50±14
CETP mass (µg/ml)	**1.0±0.2** *** **(−31%)**	1.5±0.2
CETP activity (pmol of substrate transferredper hour and µl of plasma)	**28±5** *** **(−38%)**	46±8

*** p<0.001, *p<0.05 vs controls. Numbers in parentheses denote % differences vs. controls.

### Plasma Levels of HDL Particles

**Figure 1:**
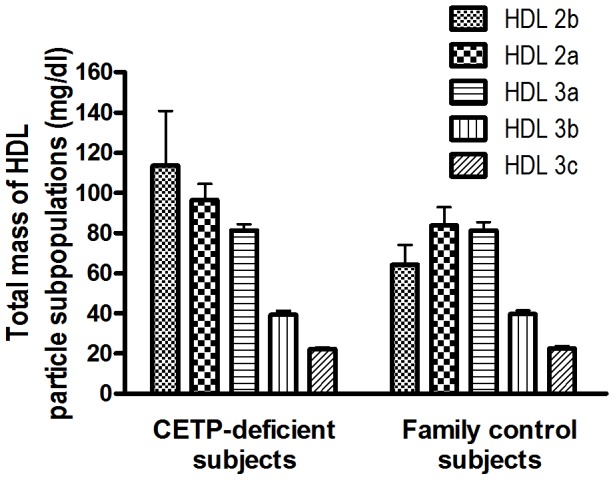
Plasma levels of HDL particle subpopulations. Total lipoprotein mass was calculated as the sum of the mass of total protein, CE, FC, PL and TG for each HDL subpopulation isolated from CETP-deficient subjects (n = 9) and from family control subjects (n = 9).

Concentrations of large HDL2 particles tended to be increased (up to 1.8-fold) in CETP-deficient subjects as compared to family controls (Fig. 1). By contrast, concentrations of small HDL3 subpopulations (Fig. 1) and those of total HDL (343±88 vs. 315±78 mg/dl respectively) did not differ between CETP-deficient subjects and controls.

### Chemical Composition of HDL Particles

Neutral lipids of the hydrophobic HDL core were strongly affected by CETP deficiency. All HDL particle subpopulations as well as total HDL from CETP-deficient subjects were significantly depleted in TG relative to HDLs from family control subjects (HDL2b, −49%, p<0.05; HDL2a, −54%, p<0.01; HDL3a, −47%, p<0.01; HDL3b, −53%, p<0.01; HDL3c, −45%, p<0.05; total HDL, −48%, p<0.01; Table 2). Moreover, CETP-deficient subjects possessed HDL2a and 3b enriched in CE (+19%, p<0.05, and +12%, p<0.05, respectively). As a result, the TG/CE ratio was reduced in each HDL subpopulation and in total HDL in CETP-deficient subjects (Table 2), 2.1-fold in HDL2b and 3a (p<0.05), 2.5-fold in HDL2a (p<0.01), 2.4-fold in HDL3b (p<0.05), 2.2-fold in HDL3c and in total HDL (p<0.05), consistent with diminished CETP activity.

**Table 2:** Chemical composition of HDL subfractions from CETP-deficient subjects (n = 9) and family control subjects (n = 9).

	Group	HDL2b	HDL2a	HDL3a	HDL3b	HDL3c	Total HDL
TG	CETP-deficient	4.3±3.0*	2.2±1.2**	2.0±1.2**	1.8±1.1**	1.8±1.2*	2.5±1.5**
	Family control	**8.5±3.8**	**4.8±1.8**	**3.8±1.1**	**3.8±1.6**	**3.3±1.8**	**4.9±1.7**
CE	CETP-deficient	28.7±3,8	26.1±4.5*	23.3±2.8	21.8±1.6*	17.8±1.0	25.3±4.0
	Family control	26.0±3.6	**21.9±3.4**	21.0±2.9	**19.4±2.2**	16.7±2.0	21.8±3.1
FC	CETP-deficient	6.4±1.4	4.1±1.1	3.1±0.6	2.5±0.4	1.7±0.5	4.3±1.5
	Family control	5.8±1.1	3.6±0.7	2.8±0.4	2.3±0.3	1.6±0.5	3.5±0.7
PL	CETP-deficient	31.8±2.5	30.2±4.2	28.8±2.0	25.6±1.1	20.5±0.9	29.1±2.2
	Family control	30.3±3.7	31.8±2.4	29.4±1.9	26.4±1.5	20.9±1.8	29.3±2.3
Protein	CETP-deficient	28.8±2.6	37.4±4.4	42.8±3.0	48.2±0.8	58.2±1.8	38.8±4.8
	Family control	29.4±1.9	37.9±3.1	43.0±2.5	48.1±1.6	57.4±2.4	40.5±3.2
A-I/A-II	CETP-deficient	29.7±4.4*	38.7±1.9 *	41.6±1.2*	47.5±3.1	47.1±1.8	3.8±0.9
	Family control	**24.3±4.0**	**36.6±1.0**	**40.3±1.0**	45.8±3.1	44.8±3.0	3.2±0.3
TG/CE	CETP-deficient	0.16±0.13*	0.09±0.06**	0.09±0.06*	0.09±0.06*	0.10±0.07*	0.11±0.07*
	Family control	**0.35±0.19**	**0.23±0.12**	**0.19±0.08**	**0.21±0.11**	**0.21±0.15**	**0.24±0.12**

TC, FC, PL and TG concentrations were measured using commercially available kits (CHOP-PAP, Biomerieux, France). Cholesteryl ester (CE) content was calculated by multiplying the difference between TC and FC by 1.67. Total protein was measured using the BCA assay. Apolipoprotein A-I and apoA-II were determined by immunonephelometry. All parameters, except the ratios, are expressed as percentage of total HDL mass. *p<0.05, **p<0.01, vs. corresponding HDL from controls.

In contrast, polar lipids of the HDL surface monolayer were less impacted by CETP deficiency (Table 2). Indeed, no systematic difference in PL content was observed between the groups; by contrast, FC tended to be elevated in HDLs from CETP-deficient subjects relative to family controls.

Finally, no difference in total protein content between the groups was observed (Table 2). Nonetheless, quantification of apoA-I and apoA-II in the protein moiety revealed a tendency to the elevation of the apoA-I content in HDLs from CETP-deficient subjects relative to the apoA-II content (Table 2). Furthermore, apoA-I content (as wt %) was significantly elevated in HDL2b, 2a and 3a in the CETP-deficient group (up to +22%, p<0.05, in HDL2b; data not shown).

### Antioxidative Activity of Small, Dense HDL

Protection of normolipidemic LDL by small, dense HDL3b and HDL3c, and by total HDL, from oxidative stress induced by an azo-initiator (AAPH) was evaluated on the basis of total HDL mass. As documented in our earlier studies [31], [33], [34], the impact of HDL on LDL oxidation is most pronounced during the propagation phase. Indeed, small, dense HDL3 from normolipidemic subjects, and particularly HDL3c, markedly prolong the propagation phase but also greatly decrease oxidation rate in this phase [31], [33], [34].

**Figure 2:**
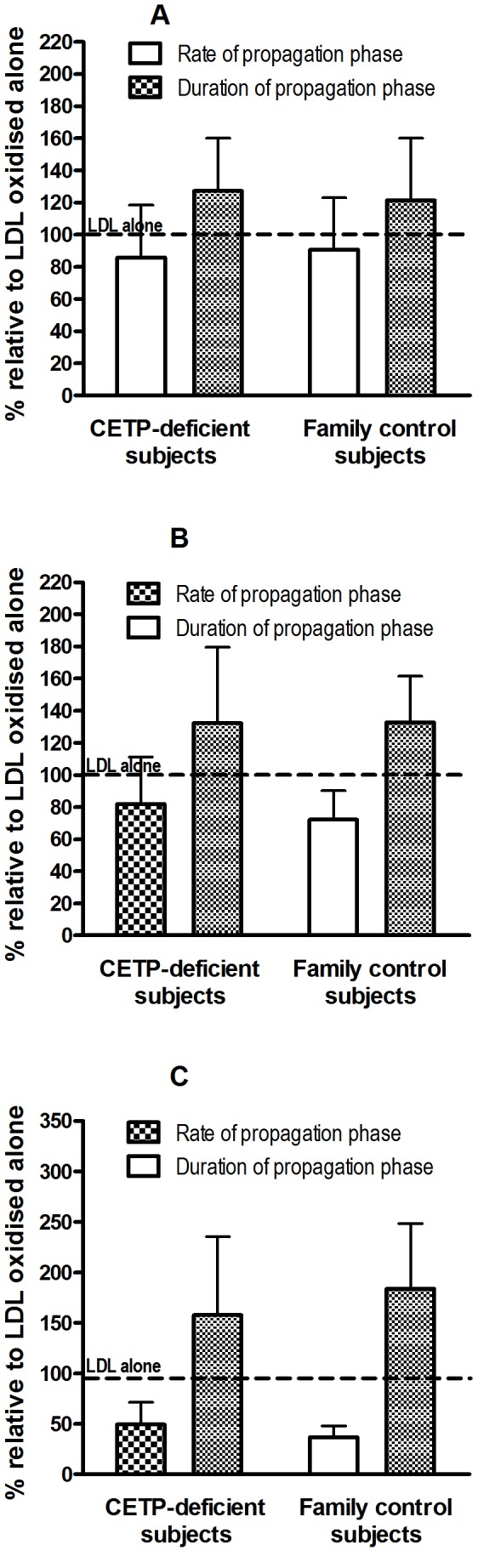
Antioxidative activity of HDL particles from CETP-deficient (n = 9) and family control (n = 9) subjects. Reference LDL (10 mg TC/dl) was incubated at 37°C in PBS in the presence of AAPH (1 mmol/l). Small, dense HDL3b (A), HDL3c (B) or total HDL (C) particles were added to LDL directly before oxidation at 10 mg total mass/dl (A, B) or 40 mg total mass/dl (C). Accumulation of conjugated dienes was continuously measured as the increment in absorbance at 234 nm; two characteristic phases were identified, the lag phase and the propagation phase. To characterise the oxidation kinetics, oxidation rate within the propagation phase and duration of this phase were calculated for each absorbance curve.

In the present study, small, dense HDL3b and 3c particles as well as total HDL from CETP-deficient subjects did not differ from the corresponding fractions isolated from family controls in terms of their capacity to protect LDL from free-radical-induced oxidation. Indeed, small, dense HDL3b (Fig. 2A) and 3c (Fig. 2B) particles as well as total HDL (Fig. 2C) from CETP-deficient subjects and from controls were of similar efficacy in prolonging the propagation phase of LDL oxidation and diminishing oxidation rate in the propagation phase of LDL.

### Levels of Systemic Oxidative Stress

CETP-deficient subjects did not reveal elevated levels of systemic oxidative stress. Indeed, plasma concentrations of both oxidised LDL (59.0±23.9 U/l for CETP-deficient subjects vs 58.4±11.5 U/l for family controls) and 8-isoprostanes (19±22 for CETP-deficient subjects vs 30±30 ng/l for family controls) as well as HDL-associated paraoxonase activity (4.69±1.00 µmol/min/mg protein for HDL3c from CETP-deficient subjects vs 4.58±1.06 µmol/min/mg protein for HDL3c from family controls) were similar between the groups.

## Discussion

We have shown that both small, dense HDL subpopulations and the total HDL fraction derived from hyperalphalipoproteinemic Dutch subjects, deficient in CETP due to a splice-site mutation in the intron 7 and premature protein truncation, display antioxidative activity towards human LDL oxidised by free radicals which is comparable to that in non-affected control members of the families. In support of these data, circulating biomarkers of systemic oxidative stress (oxidised LDL and 8-isoprostanes) did not differ between CETP-deficient subjects and age-matched normolipidemic family controls.

Mechanisms of HDL-mediated protection of LDL from free-radical-induced oxidation involve inactivation of lipid hydroperoxides (LOOHs) *via* a two-step mechanism which includes transfer of LOOHs to HDL, modulated by physical properties of the surface lipid monolayer, with subsequent reduction to redox-inactive hydroxides by Met residues of apoA-I [35], [36], [37], [38], [39]. The content and composition of surface lipids and apoA-I therefore represent key compositional determinants of the antioxidative properties of HDL. In our studies, HDL particles from carriers revealed pronounced compositional changes at the level of their lipid core, including enrichment in CE (up to +19%, p<0.05) and depletion of TG (up to −54%, p<0.01) with a reduced TG/CE ratio (up to 2.5-fold, p<0.01); surface lipids (PL, FC) however remained unaffected. In parallel, HDL content of apoA-I was increased in HDLs from carriers (up to +22% vs. controls, p<0.05). In clear contrast, our earlier studies revealed that HDL particles were depleted of CE and of apoA-I and enriched in TG in atherogenic dyslipidemia of insulin-resistant states associated with elevated CETP activity, such as metabolic syndrome and Type 2 diabetes; such compositional alterations were paralleled by reduced antioxidative activity of HDL [40]. Similar structure-functional relationships were documented in normotriglyceridemic low HDL-C dyslipidemia [41]. Compositional modifications of HDLs observed in CETP-deficient subjects in our present study are therefore inconsistent with defective antioxidative activities.

Metabolically, reduced CETP activity in heterozygous CETP deficiency modifies core lipid composition of HDL particles, delaying their catabolism and increasing their apoA-I content. Functionality of HDL which is largely determined by the content of apoA-I, the key component underlying antioxidative activities of HDL [42], can therefore be potentially improved by CETP deficiency. On the other hand, prolonged HDL lifespan in CETP-deficient subjects suggests the possibility of enhanced apoA-I oxidation [43] which may result in reduced HDL capacity to protect LDL from free radical-induced oxidation secondary to the reduced apoA-I content of redox-active Met residues [42]. The relevance of the latter pathway is however in disagreement with our present data which support the contention that HDL exhibits antioxidative activity within the normal range in heterozygous CETP deficiency. Consistent with the data derived from studies focussing on extreme genetics and common variants [13], [16], [44], [45], HDL-C levels were increased 1.5-fold, and CETP mass and activity were decreased by −31% and −38% respectively, in carriers versus non-carriers in our present study. Such consistency in biomarkers of lipid metabolism between earlier and present studies suggests the pertinence of our present findings to the general context of heterozygous CETP deficiency.

Our conclusion regarding the normal antioxidative function of HDL in heterozygous CETP deficiency is further supported by published data on antioxidative activity of HDL particles in CETP-deficient states. Indeed, the capacity of HDL to protect LDL against oxidative modification is not affected by CETP overexpression in a transgenic mice model [46]. Furthermore, the CETP inhibitor dalcetrapib decreased circulating levels of oxidised LDL in familial hypoalphalipoproteinemia [47], whereas CETP inhibition in vitro by a monoclonal antibody renders LDL more resistant to oxidation [48], observations which are consistent with the absence of a deficiency in the HDL-mediated protection of LDL from oxidation in vivo. Importantly, the normal capacity of HDL to protect LDL from oxidative modification by free radicals in the arterial intima can contribute to protection from atherogenesis. Indeed, despite the fact that the pathophysiological importance of LDL oxidation by free radicals remains to be demonstrated, this pathway is operative in human atherosclerosis and can be efficiently inhibited by HDL [49], [50], [51].

Normal antioxidative activity of HDL in CETP-deficient subjects as observed by us is also important in the context of the role of CETP for the production of small HDL particles. Indeed, recycling of lipid-free/lipid-poor apoA-I from mature HDL combined with the subsequent actions of hepatic lipase and scavenger receptor class B type I (SR-BI) constitutes an important aspect of the lipoprotein-remodelling action of CETP [52]. As small, dense HDL particles display potent anti-atherogenic activities [31], [53], [54], CETP deficiency might theoretically impair atheroprotective properties of the total plasma HDL pool via reduction in plasma levels of small HDL; however, our present data do not support such a hypothesis. The observation of normal antioxidative properties of total HDL is further consistent with the minor contribution of large, light HDL2 to this biological activity as reported earlier [31]. Indeed, although the composition of large, light HDL2 particles was strongly affected by CETP deficiency in our studies, the antioxidative properties of total HDL remained unchanged.

Importantly, CETP deficiency does not compromise the capacity of HDL to efflux cellular cholesterol, another key atheroprotective activity of HDL. Indeed, homozygous CETP deficiency features accumulation of large HDL2 particles with elevated content of apoE and lecithin:cholesterol acyltransferase (LCAT); such particles display elevated cholesterol efflux capacity on THP-1 cells [21]. In addition, HDLs isolated from a compound heterozygote for a known D459G variant and a novel 18-bp deletion mutation in the CETP promoter display elevated cholesterol efflux capacity via SR-BI [22]. Together, these considerations further support the concept that partial CETP deficiency does not impair functionality of plasma HDL [55].

In order to evaluate the potential physiopathological and therapeutic relevance of this conclusion, the relationship between CETP deficiency and cardiovascular risk should be taken into account. Mutations in the CETP gene frequently cause familial hyperalphalipoproteinemia [56]; most CETP polymorphisms, such as three common CETP genotypes Taq1B (+279G>A), Ile405Val (+16A>G) and −629C>A, are equally associated with elevated HDL-C levels [13], [16], [44], [45]. Potentially reflecting the atheroprotective role of HDL, the TaqIB, I405V and −629C>A genotypes associated with lower CETP activity and higher HDL-C levels are inversely associated with coronary risk [13], [14], [16]. Moreover, individuals with longevity syndrome display an elevated frequency of the B2 allele of TaqIB polymorphism and of the Int 14A variant in the CETP gene, both associated with high levels of HDL-C [57], [58].

These results support the hypothesis of a causal relationship between low CETP activity, high HDL-C and reduced cardiovascular risk, which forms a basis for therapeutic strategies involving partial CETP inhibition in humans [6]. Indeed, metabolic conditions of CETP inhibition share some similar characteristics to those of CETP deficiency, the latter involving reduced levels of CETP protein; however, it is essential to emphasise that the two metabolic conditions cannot be directly compared. Studies in rabbits, a species with naturally high levels of CETP, support the therapeutic potential of partial CETP inhibition as an approach to retarding atherogenesis [59], [60]. CETP inhibitors are presently the most potent HDL-raising agents, with dose-dependent HDL-C elevation of up to +100% or more for some agents [6]. HDL particles which are functional in terms of their capacity to efflux cellular cholesterol are formed following either potent or moderate levels of CETP inhibition by anacetrapib [61] or dalcetrapib [62], [63]. As a result, the net action of partial CETP inhibition on reverse cholesterol transport from peripheral tissues to the liver is not deleterious in CETP-expressing species. Detrimental effects of CETP inhibition on atheroprotective functions of HDL other than cholesterol efflux cannot however be excluded [64] and remain in the focus of ongoing discussions, in view of further clinical development of other CETP inhibitors [43], [65], [66]. Our present results in partial genetic CETP deficiency shed a new light on this controversial area, suggesting that moderate CETP inhibition should not exert deleterious impact on the antioxidative properties of HDL.
